# Dominant Negative Phenotype of *Bacillus thuringiensis* Cry1Ab, Cry11Aa and Cry4Ba Mutants Suggest Hetero-Oligomer Formation among Different Cry Toxins

**DOI:** 10.1371/journal.pone.0019952

**Published:** 2011-05-16

**Authors:** Daniela Carmona, Claudia Rodríguez-Almazán, Carlos Muñoz-Garay, Leivi Portugal, Claudia Pérez, Ruud A. de Maagd, Petra Bakker, Mario Soberón, Alejandra Bravo

**Affiliations:** 1 Departmento de Microbiologia Molecular, Instituto de Biotecnología, Universidad Nacional Autónoma de México, Cuernavaca, Morelos, Mexico; 2 Bioscience, Plant Research International, Wageningen, The Netherlands; Cinvestav, Mexico

## Abstract

**Background:**

*Bacillus thuringiensis* Cry toxins are used worldwide in the control of different insect pests important in agriculture or in human health. The Cry proteins are pore-forming toxins that affect the midgut cell of target insects. It was shown that non-toxic Cry1Ab helix α-4 mutants had a dominant negative (DN) phenotype inhibiting the toxicity of wildtype Cry1Ab when used in equimolar or sub-stoichiometric ratios (1∶1, 0.5∶1, mutant∶wt) indicating that oligomer formation is a key step in toxicity of Cry toxins.

**Methodology/Principal Findings:**

The DN Cry1Ab-D136N/T143D mutant that is able to block toxicity of Cry1Ab toxin, was used to analyze its capacity to block the activity against *Manduca sexta* larvae of other Cry1 toxins, such as Cry1Aa, Cry1Ac, Cry1Ca, Cry1Da, Cry1Ea and Cry1Fa. Cry1Ab-DN mutant inhibited toxicity of Cry1Aa, Cry1Ac and Cry1Fa. In addition, we isolated mutants in helix α-4 of Cry4Ba and Cry11Aa, and demonstrate that Cry4Ba-E159K and Cry11Aa-V142D are inactive and completely block the toxicity against *Aedes aegypti* of both wildtype toxins, when used at sub-stoichiometric ratios, confirming a DN phenotype. As controls we analyzed Cry1Ab-R99A or Cry11Aa-E97A mutants that are located in helix α-3 and are affected in toxin oligomerization. These mutants do not show a DN phenotype but were able to block toxicity when used in 10∶1 or 100∶1 ratios (mutant∶wt) probably by competition of binding with toxin receptors.

**Conclusions/Significance:**

We show that DN phenotype can be observed among different Cry toxins suggesting that may interact *in vivo* forming hetero-oligomers. The DN phenotype cannot be observed in mutants affected in oligomerization, suggesting that this step is important to inhibit toxicity of other toxins.

## Introduction

*Bacillus thuringiensis* (*Bt*) bacteria produce insecticidal crystal (Cry) proteins that are used in the control of insect pests important for agricultural crops and against vectors of human diseases [1]. Cry toxins have been characterized as pore-forming toxins. Their mechanism of action involves specific interactions with several receptors and insertion of part of the toxin into the apical membrane of insect midgut cells, forming pores that finally kill the larvae [2]. It was shown that Cry1A binding to cadherin receptor induced the cleavage of an amino-terminal region including helix α-1 leading to toxin oligomerization. Cry oligomers bind to aminopeptidase or alkaline phosphatase receptors [3], [4], and inserts into the membrane to form toxic pores [1], [2]. Helix α-3 of Cry toxin is involved in toxin oligomerization [5] and helix α-4 in membrane insertion and pore formation [6]. Mutants affected in helices α-3 or α-4 are thus affected in pore formation activity and completely lost toxicity against their target insect [5]–[7].

Recently it was reported that some non-toxic Cry1Ab helix α-4 mutants showed a dominant negative (DN) phenotype, since they inhibited wildtype insecticidal activity at substochiometric ratios [8]. These mutants were able to form homo-oligomers but were affected in their pore formation activity and it was proposed that monomers of the Cry1Ab-DN mutants are able to form oligomeric structures with wildtype Cry1Ab functioning as effective antitoxins that block toxicity of the wildtype toxin and then have the potential to be use to protect special ecosystems from the potential effects of Cry toxins on non-target insects [8]. Similar antitoxins from different pore forming toxins that affect mammalian cells as protective antigen PA subunit of anthrax toxin from *Bacillus anthracis* [9], [10], ClyA Cytotoxin from *Escherichia coli* [11] and vacuolating toxin VacA from *Helicobacter pylori* [12] have also been reported. DN inhibitors of PA, ClyA and VacA, are inactive mutant-toxins that are able to form oligomer structures but are affected in their pore formation activity. It was suggested that they work as powerful inhibitors since they are able to co-assemble into oligomers together with their corresponding wildtype toxin resulting in an effective inactivation of pore formation activity of their wildtype toxins resulting in the complete loss of toxicity [9]–[12]. In addition a dominant-negative mutant of HCN channels present in the ventricular myocardium were also reported. The nonfunctional HCN2 mutations affected pore formation of the channel and suppressed HCN2 wildtype activity in a dominant-negative manner [13]. In all these reports, the DN phenotype has been regarded as compelling evidence for *in vivo* oligomer formation and for this reason they were proposed as anti-toxins to control the diseases that these bacterial-toxins induce in mammalian organisms or to control arrhythmogenesis and cardiac pacing [9]–[13].

*Bt* subsp. *israelensis* produce different Cry toxins (Cry4Aa, Cry4Ba, Cry10Aa and Cry11Aa) that are active against mosquito larvae [14]. It was proposed that mosquitocidal Cry toxins share a similar mechanism of action with Cry1A toxins, that are active against lepidopteran insect pests, since similar Cry-binding molecules have been identified in mosquitoes, including cadherin [15], [16], aminopeptidase [17], [18], and alkaline phosphatase [19]. Interestingly, several reports show a synergism between Cry11Aa and Cry4Ba although the molecular mechanism of this synergistic effect remains unknown [20]–[22]. Cry11A and Cry4Ba mutants in helix α-4 were affected in toxicity against mosquito larvae [23], [24].

In this work we used the previously described non-toxic Cry1Ab-D136N/T143D mutant to analyze if its DN phenotype extends to other Cry1 toxins. Cry1Ab-D136N/T143D was a potent inhibitor of Cry1Ab *in vivo* and was affected in pore formation but not in toxin-oligomerization [8]. We analyzed if this DN-mutant was able to inhibit the toxicity of the highly related Cry1Aa and Cry1Ac toxins and also of other Cry1 toxins that are active against *M. sexta* (Cry1Ca, Cry1Da, Cry1Ea and Cry1Fa). In addition we isolated helix α-4 mutants of Cry4Ba and Cry11Aa and analyzed their DN phenotype with both wt Cry4Ba and Cry11Aa toxins. Our results show that Cry1Ab DN mutant functions as antitoxin of Cry1Aa, Cry1Ac and Cry1Fa, while Cry11Aa and Cry4Ba DN mutants inhibit the toxicity of both Cry11Aa and Cry4Ba toxins. These results suggest that in some cases Bt Cry toxins have the potential to form hetero-oligomers.

## Results

### In vivo inhibition of toxin insecticidal activity

**Figure 1:**
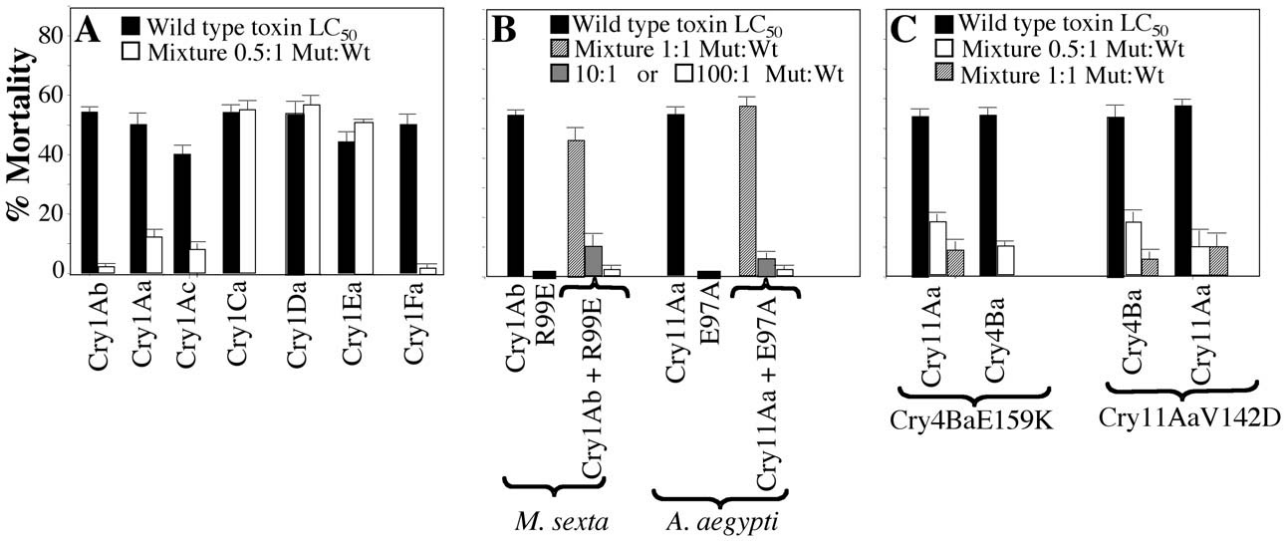
*In vivo* analysis of the Dominant Negative phenotype of Cry mutants. Panel A, Toxicity assays against *Manduca sexta* larvae of different Cry toxins at their corresponding LC_50_ concentration (see table 1) (black bars) and mixtures of Cry1Ab-D136N/T143D mutant with these wildtype Cry toxins in a protein ratio 0.5∶1 (mutant∶wildtype) (white bars). Panel B, Toxicity assays against *Manduca sexta* or *Aedes aegypti* larvae of Cry1Ab or Cry11Aa at their corresponding LC_50_ concentration (black bar) or in a mixture with Cry1Ab-R99E or Cry11Aa-E97A mutant toxins in a protein ratio 1∶1 (dashed bars), 10∶1 (mutant∶wildtype) (grey bars) or 100∶1 (mutant∶wildtype) (white bars). Panel C, Toxicity assays against *Aedes aegypti* larvae of Cry4Ba or Cry11Aa at their corresponding LC_50_ concentration (black bar) or in a mixture with mutant toxins in a protein ratio 0.5∶1 (mutant∶wildtype) (white bars) or 1∶1 (dashed bars).

To compare the potency of the Cry1Ab helix α-4 mutant D136N/T143D as DN inhibitor of other Cry1 toxins, we tested its ability to inhibit the toxicity of other Cry1 toxins that are also active against *M. sexta* such as Cry1Aa, Cry1Ac, Cry1Ca, Cry1Da, Cry1Ea and Cry1Fa. We first determined the medium lethal concentration (LC_50_ value) of each protoxin performing bioassays against first instar *M. sexta* larvae using a series of different protoxin concentrations (Table 1). Then we fed *M. sexta* larvae with mixtures of Cry1Ab-D136N/T143D mutant with the different wildtype Cry protoxins (at their corresponding LC_50_ concentration) in a protein ratio 0.5∶1 (mutant∶wildtype). Figure 1A shows that Cry1Ab-D136N/T143D mutant blocked the action of Cry1Aa, Cry1Ac and Cry1Fa toxins, but did not affect the toxicity of Cry1Ca, Cry1D or Cry1Ea. The inhibition of toxicity of Cry1Aa, Cry1Ac and Cry1Fa toxins by Cry1Ab-D136N/T143D mutant was observed at sub-stoichiometric ratio.

**Table 1:** Toxicity of Cry toxins.

Protoxin	LC_50_(95% fiducial limits)	Target insect
Cry1Aa	3.7 (2.8–4.7)^a^	*Manduca sexta*
Cry1Ab	2.9 (1.8–4.8)^a^	*Manduca sexta*
Cry1Ac	1.8 (2.0–3.6)^a^	*Manduca sexta*
Cry1Ca	52.5 (41.6–75.5)^a^	*Manduca sexta*
Cry1Da	25.1 (19.9–44.1)^a^	*Manduca sexta*
Cry1Ea	225.0 (190.1–280.8)^a^	*Manduca sexta*
Cry1Fa	14.1 (8.2–22.3)^a^	*Manduca sexta*
Cry4Ba	83.7 (55.4–130.2)^b^	*Aedes aegypti*
Cry4Ba L152D	108.1 (79.2–190.4)^b^	*Aedes aegypti*
Cry4Ba-E159K	>2,000^b^	*Aedes aegypti*
Cry11Aa	454.11 (312.7–760.6)^b^	*Aedes aegypti*
Cry11Aa-V142D	>2,000^b^	*Aedes aegypti*

a ng/cm^2^;

b ng/ml.

We analyzed if the DN phenotype could be observed with a different inactive mutant affected in oligomerization such as the Cry1Ab-R99A located in helix α-3 of domain I [5]. Figure 1B shows that this mutant did not have a DN phenotype, since it was unable to inhibit the toxicity of the wildtype toxin when used a sub-stoichiometric or equimolar ratios. In contrast, when we tested higher concentrations of the Cry1Ab-R99A mutant, up to 10 or 100 fold higher concentration of the mutant than the wildtype toxin; we found that Cry1Ab-R99A was able to inhibit toxicity of wildtype toxin (Fig. 1B). This is probably due to binding competition for toxin receptors since mutation of Cry1Ab-R99A is located in helix α-3, a region that is not involved in toxin interaction with receptors. These data suggest that binding competition requires much higher concentrations of the competitor, indicating a complete different mechanism than DN phenotype.

### Isolation of Cry4Ab and Cry11Aa mutants in helix α-4

**Figure 2:**
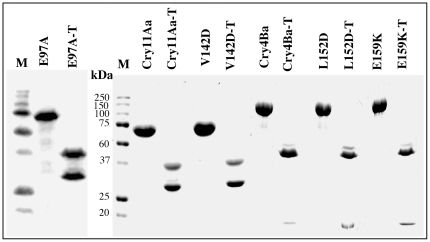
Analysis of Cry4Ba and Cry11Aa proteins in SDS-PAGE. Protoxins and trypsin-activated proteins were stained with Coomassie blue. Molecular weight markers used in all SDS-PAGE were precision pre-stained plus standards all blue (BioRad, Hercules CA). Soluble protoxins are Cry11Aa, E97A, V142D, Cry4Ba, L152D and E159K. Trypsin activated toxins are Cry11Aa-T, E97A-T, V142D-T, Cry4Ba-T, L152D-T and E159K-T.

As mentioned previously the mosquitocidal Cry11Aa and Cry4Ba have a synergistic effect when fed together to certain mosquito larvae [20]–[22]. In order to analyze if these toxins have a similar mechanism of action than Cry1A toxins, we isolated four mutants in helix α-4 of Cry4Ba (A145C, L152D, E159K and R158A) and three mutants in Cry11Aa (N128D, Q135C and V142D). Some of these mutants were highly susceptible to trypsin degradation (Cry4Ba-A145C, Cry4Ba -R158A, Cry11Aa-N128D and Cry11Aa-Q135C) and were not further analyzed. The crystal inclusions of Cry4Ba-L152D, Cry4Ba-E159K and Cry11Aa-V142D mutants were purified and protoxins activated with trypsin, as described in materials and methods. Figure 2 shows the SDS-PAGE electrophoretic profile of protoxins and activated toxins of Cry4Ba and Cry11Aa mutant toxins analyzed in this work. The Cry4Ba mutants produced a similar 40 and 18 kDa activated toxin fragment as the wildtype and the Cry11Aa mutant also showed a similar activation profile of 36 and 32 kDa as the Cry11Aa, indicating no major effects in toxin structure stability. The insecticidal activity of these wildtype and mutant proteins was analyzed in bioassays against *Aedes aegypti* larvae as described, using purified crystal suspensions that were sonicated to avoid aggregation [25]. The Cry4Ba showed a lower LC_50_ value than Cry11Aa toxin (Table 1). The Cry4Ba-L152D mutant was active against *A. aegypti* while the mutants Cry4Ba-E159K and Cry11Aa-V142D were inactive (Table 1). To test the ability of Cry4Ba-E159K and Cry11Aa-V142D to inhibit the toxicity of their corresponding wildtype toxins we used an equimolar (1∶1) as well as a lower ratio (0.5∶1) of mutant∶wildtype to fed the mosquito larvae. Figure 1C shows that both mutant toxins were able to block the action of their corresponding wildtype toxin showing a DN phenotype since inhibition was observed at sub-stoichiometric ratios. We finally analyzed if Cry4Ba-E159K was able to inhibit the toxicity of Cry11Aa and if Cry11Aa-V142D could affect toxicity of Cry4Ba against *A. aegypti*. Figure 1C shows that these mutants blocked toxicity of a different wildtype toxin suggesting that *in vivo* Cry11Aa and Cry4Ba toxins may interact in *A. aegypti*. However, when we used the previously described non-toxic Cry11Aa-E97A mutant, located in helix α-3 that is affected in oligomerization [26], we found that this mutant did not show a DN phenotype since it was unable to inhibit the toxicity of the wildtype toxin when tested at 0.5∶1 or 1∶1 ratios. Similarly to the data of Cry1Ab-R99A mutant described above, we found that Cry11Aa-E97A mutant was able to block toxicity at 10∶1 or 100∶1 ratios (mutant∶wt) suggesting that this inhibition is due to a different mechanism, most probably involving binding competition.

## Discussion

Inactive mutants of PA subunit of the anthrax toxin were among the first reports of DN mutants in pore forming toxins. These mutants were able to form oligomer structures but were affected in their pore formation activity, working as powerful inhibitors of anthrax toxin since they co-assemble into hetero-oligomers with the wildtype toxin resulting in an effective inactivation of pore formation and toxicity [9], [10]. Similar results were obtained with other pore forming toxins such as VacA and ClyA, and in HCN ionic channels, where mutants affected in pore formation but not in oligomerization also showed a DN phenotype [11]–[13]. It was proposed that the underlying mechanism involves the hetero-oligomerization between the mutant toxins with the wildtype resulting in a DN effect, blocking the wildtype toxicity [9]–[13].

The mutant Cry1Ab-D136N/T143D in helix α-4, analyzed in this study, was previously shown not to impair toxin assembly in a pre-pore structure, but rather to block an essential conformational transition of the assembled complex necessary for membrane insertion and pore formation [8]. It was reported that this mutant inhibited the activity of wildtype Cry1Ab *in vivo* against *M. sexta* larvae and *in vitro* by analyzing pore formation in black lipid bilayers [8].

In this work we hypothesized that if the DN phenotype of Cry1Ab-D136N/T143D extends to different Cry toxins this could suggests hetero-oligomerization among different Cry toxins. We thus analyzed if the Cry1Ab-D136N/T143D mutant was capable of blocking the toxicity of other Cry1 toxins when mixed at sub-stoichiometric ratios.

Our data show that DN mutant of Cry1Ab was able to inhibit toxicity of Cry1Aa, Cry1Ac and Cry1Fa toxins at 0.5∶1 ratio (mutant∶wt). These data suggest that the molecular mechanism observed in DN phenotype involves co-assembling between different Cry toxin-monomers forming hetero-oligomeric structures. In addition, we isolated mutants in helix α-4 of Cry4Ba and Cry11Aa toxins that are specific against mosquitoes. Some of these mutants lost toxicity and also showed a clear DN phenotype when mixed with their corresponding wildtype toxin. The DN phenotype was also observed between the Cry4Ba-E159K mutant and Cry11Aa or the Cry11Aa-V142D mutant and Cry4Ba wildtype suggesting interaction between these two toxins. These data resulted quite interesting since it was reported that Cry11Aa and Cry4Ba have synergistic activity [19]–[21] showing in some cases up to 10 fold higher activity in the mixture than the expected mortality from the individual toxins.

Recently, a 3D-structure model of the Cry4Aa pre-pore oligomer was published, based in the structure of the trimeric unit cell of the X-ray crystal structure of the Cry4Ba toxin [27]. In this structural model the authors proposed that the Cry4Aa oligomer is stabilized by some residues of helices α-3, α-4 and α-6 and that pore formation may involved and insertion into the lipid bilayer of the hairpin conformed by helices α-4 and α-5 of domain I, while domains II and III remain in the membrane surface [27]. Comparison of amino acid sequences of helices α-3, α-4 and α-6 among the different Cry toxins used in this study did not revealed any apparent explanation for the specificity of the DN phenotype, indicating that primary sequence of these domain I regions do not determine the binding interaction among different Cry toxins and/or that other toxin regions are involved in this interactions, this remains to be analyzed.

The role of toxin oligomerization in inducing the DN phenotype was supported by the fact that Cry1Ab-R99A or Cry11Aa-E97A mutants affected in the process of oligomerization [5], [26] did not induce a DN response since they were unable to inhibit toxicity of their corresponding wildtype toxin at sub-stoichiometric ratios neither at equimolar ratios. These data strongly suggest that oligomerization among DN mutants and wildtype monomers, represents the mechanism responsible of the DN phenotype.

Our results show that helix α-3 mutants, Cry1Ab-R99A or Cry11Aa-E97A, do not have a DN phenotype but inhibited toxicity of their corresponding wt toxin when tested at higher ratios such as 10∶1 or 100∶1 (mutant∶wildtype), suggesting competition for receptor binding. These data are similar to some reported mutants of the anthrax toxin, since a PA mutant affected in toxin oligomerization did not show a DN phenotype since it was unable to form hetero-oligomers with the wildtype toxin [28]. However, this mutant still bound to, and competed, receptor binding causing a competitive inhibition of anthrax toxin action at 10∶1 ratio [28].

Although the physiological role of hetero-oligomerization among different toxins remains to be analyzed, it is interesting to note that synergism between different Cry toxins such as Cry4Ba and Cry11Aa against mosquito larvae have been reported [20]–[22]. The synergism between Cry1A toxins has been also documented before. Specifically, Cry1Ab and Cry1Ac synergized against *Chilo partellus* larvae showing up to five fold higher activity when both toxins are present in the bioassay [29]. Also it was reported that Cry1Aa and Cry1Ac have a synergistic effect against *Lymantria dispar* larvae, increasing their toxicity up to 4.9 fold and in this case voltage clamping assays demonstrated that combination of both toxins resulted in a greater pore formation activity than the individual toxins [30]. In the case of Cry1A and Cry1Fa, there are no data of synergism between them. However, when both toxins were expressed in Bt-cotton plants, the control of *Helicoverpa zea* and *Spodoptera* species was more effective than single protein Bt-cotton plants [31]. Overall, these data may suggest that synergistic effect could be related to the interaction of different Cry toxins, forming a complex that is more effective in killing the target larvae. Most Bt isolates produce more than one Cry toxin, thus, it is tempting to speculate that hetero-oligomerization of Cry toxins could have been selected in nature as a mechanism to modulate toxicity and insect specificity of these family of pore-forming toxins.

## Materials and methods

### Construction of Cry4Ba and Cry11Aa mutants

Mutants in helix α-4 of Cry4Ba (A145C, L152D, E159K and R158A) and Cry11Aa (N128D, Q135C and V142D) were produced by site-directed mutagenesis (Quick-Change, Stratagene, La Jolla, CA) using pCG6 plasmid [32] containing *cry11Aa* gene or pHT611 plasmid [33] harboring the *cry4Ba* gene as templates. Appropriate oligonucleotides were synthesized for each mutant. Automated DNA sequencing at Instituto de Biotecnología-UNAM's facilities verified the single point mutations. Acrystalliferous Bt strain 407 was transformed with recombinant plasmids as reported [34] and selected in Luria broth at 30°C supplemented with 10 µg ml^−1^ erythromycin.

### Purification of Cry1, Cry4Ba and Cry11Aa toxins

The inclusion bodies of Cry1C, Cry1Da, Cry1Ea and Cry1Fa protoxins were obtained as recombinant proteins expressed in *Escherichia coli* as reported [35]. Cry1Aa, Cry1Ab and Cry1Ac were produced in *Bt* bacteria as described [36]. Protoxins were solubilized in alkaline buffer 50 mM Na_2_CO_3_, 0.2% β-mercaptoethanol, pH 10.5.

Bt transformant strains of Cry4Ba and Cry11Aa proteins were grown at 30°C in nutrient broth sporulation medium with erythromycin until complete sporulation. Crystal inclusions were purified by sucrose gradients [37]. Cry4Ba protoxin was solubilized in alkaline buffer and activated with trypsin in a mass ratio of 1∶20 w/w trypsin/protoxin, for 4 h at 37°C. Cry11Aa was solubilized in 100 mM NaOH, 1 h at 4°C. The pH was equilibrated at pH 8.6 with same volume of 1M Tris HCl pH 8 and activated with trypsin (1∶50 w/w trypsin/protoxin) for 2 h at 25°C. Phenylmethylsulfonyl-fluoride (1 mM final concentration) was added to stop proteolysis. Finally proteins were visualized in SDS-PAGE gels stained with Coomassie blue. Molecular weight markers were precision pre-stained plus standards all blue (BioRad, Hercules CA).

### Bioassays

Bioassays of Cry1A protoxins were performed with first instar *M. sexta* larvae. Soluble protoxins (from 0.1 to 2000 ng/cm^2^) were applied onto the diet surface of 24-well plates, using 24 larvae per toxin concentration in triplicate. Protein was determined by the Bradford assay. Mortality was recorded after seven days and lethal concentration (LC_50_) value in ng of toxin per cm^2^ of diet was estimated by Probit (Polo-PC LeOra Software).

The bioassays of Cry4Ba and Cry11Aa against forth instar *A. aegypti* larvae were done with purified crystal suspensions that were sonicated to avoid aggregation [25]. Ten different concentrations of purified crystals were used (50–6000 ng/mL) in 100 mL of dechlorinated water containing 20 early fourth-instar larvae. Negative control (dechlorinated water) was included in the bioassay, and the viability of larvae was examined after 24 h. The mean lethal concentration (LC_50_) was estimated as described above. We used crystal suspensions due to the feeding behavior of these larvae since they fed by filtering and then a soluble protoxin is not toxic.

For DN assays different ratios of mutant∶wildtype (0.5∶1, 1∶1, 10∶1 and 100∶1; w∶w) were assayed. The concentration of wildtype toxins used in DN-bioassays corresponds to the LC_50_ value of each wt toxin as show in table 1.
